# RNA-Seq Analysis of the *Sclerotinia homoeocarpa* – Creeping Bentgrass Pathosystem

**DOI:** 10.1371/journal.pone.0041150

**Published:** 2012-08-08

**Authors:** Angela M. Orshinsky, Jinnan Hu, Stephen O. Opiyo, Venu Reddyvari-Channarayappa, Thomas K. Mitchell, Michael J. Boehm

**Affiliations:** 1 Department of Plant Pathology, The Ohio State University, Columbus, Ohio, United States of America; 2 Molecular and Cellular Imaging Center-Columbus. The Ohio State University, Columbus, Ohio, United States of America; 3 Dale Bumpers National Rice Research Center, Stuttgart, Arizona, United States of America; University of Georgia, United States of America

## Abstract

*Sclerotinia homoeocarpa* causes dollar spot disease, the predominate disease on highly-maintained turfgrass. Currently, there are major gaps in our understanding of the molecular interactions between *S. homoeocarpa* and creeping bentgrass. In this study, 454 sequencing technology was used in the *de novo* assembly of *S. homoeocarpa* and creeping bentgrass transcriptomes. Transcript sequence data obtained using Illumina's first generation sequencing-by-synthesis (SBS) were mapped to the transcriptome assemblies to estimate transcript representation in different SBS libraries. SBS libraries included a *S. homoeocarpa* culture control, a creeping bentgrass uninoculated control, and a library for creeping bentgrass inoculated with *S. homoeocarpa* and incubated for 96 h. A Fisher's exact test was performed to determine transcripts that were significantly different during creeping bentgrass infection with *S. homoeocarpa*. Fungal transcripts of interest included glycosyl hydrolases, proteases, and ABC transporters. Of particular interest were the large number of glycosyl hydrolase transcripts that target a wide range of plant cell wall compounds, corroborating the suggested wide host range and saprophytic abilities of *S. homoeocarpa*. Several of the multidrug resistance ABC transporters may be important for resistance to both fungicides and plant defense compounds. Creeping bentgrass transcripts of interest included germins, ubiquitin transcripts involved in proteasome degradation, and cinnamoyl reductase, which is involved in lignin production. This analysis provides an extensive overview of the *S. homoeocarpa*-turfgrass pathosystem and provides a starting point for the characterization of potential virulence factors and host defense responses. In particular, determination of important host defense responses may assist in the development of highly resistant creeping bentgrass varieties.

## Introduction

*Sclerotinia homoeocarpa* (Bennett) is an ascomycete fungus that causes dollar spot disease on turfgrass world-wide [1]. *S. homoeocarpa* can affect all species of turfgrass as well as some dicot plants [1]. Creeping bentgrass (*Agrostis stolonifera* L.) is a cool-season turfgrass that is common on golf course greens in the United States and Canada [2]. Many commonly used creeping bentgrass cultivars are highly susceptible to dollar spot disease and are subject to frequent, low mowing practices that promote disease outbreaks [1], [3].

Symptoms of dollar spot include straw-colored, hourglass-shaped lesions with characteristic reddish-brown borders. Diseased areas grow to about 2.5 cm wide [1]. Under high disease pressure and favorable conditions the diseased areas will merge to form larger patches of diseased turf. Environmental conditions favoring disease development have been well documented [4], [5], [6]; however, prediction models designed to reduce fungicide inputs have been ineffective due to a lack of understanding of the *S. homoeocarpa* lifestyle, epidemiology, and disease etiology [1], [4].

*S. homoeocarpa* is thought to overwinter in the thatch of turf swards as stroma. In the spring, *S. homoeocarpa* becomes active and infects the newly emerging leaf tissue through wounds, stomates, and directly with appressorium formation [7], [8], [9]. Only infertile apothecia have been recorded for North American isolates [10]; however, population studies suggest that genetic recombination in this fungus is possible [11]. Therefore, the possibility of ascospore production and the role of sexual or asexual spores as initial inoculum for dispersal of *S. homoeocarpa* cannot be discounted. Early studies noted root browning and cell death through the production of diffusible toxins [12]. Recent studies have identified several tetranorditerpenoid compounds that could be responsible for the root-browning and exhibit extremely phytotoxic properties; however, a correlation between the production of these compounds and disease symptoms was not established [13].

Cultural management strategies for dollar spot include maintaining adequate nitrogen balance, promotion of air flow to assist in dew removal, and using moderately resistant cultivars or species of turf [14], [15]. On highly maintained areas such as golf courses, cultural practices are not sufficient for management of dollar spot, and fungicides are often applied biweekly to weekly. High amounts of fungicide use have resulted in resistance to several chemical classes commonly used on turf [1]. Other nonfungicide products that have been marketed for dollar spot control include plant defense activators that work by activating two different plant defense pathways: induced systemic resistance (ISR) and systemic acquired resistance (SAR). However, it is unclear which pathway would be most beneficial for preventing dollar spot epidemics.

A better understanding of the molecular interactions between *S. homoeocarpa* and creeping bentgrass will be essential for the development of more sustainable and practical management strategies, including the use of plant defense activators and the development of cultivars with increased resistance to *S. homoeocarpa*.

The introduction of next generation sequencing, also termed massively parallel sequencing (MPS), has enabled researchers to sequence the genomes and transcriptomes of organisms at a relatively low cost in return for a vast amount of data with quantitative properties [16], [17], [18], [19]. In this paper, two MPS technologies were used to generate sequence data for RNA-Sequence (RNA-Seq) analysis: Illumina's sequencing-by-synthesis (SBS) and Roche's 454-pyrosequencing. The 454 reads were used for the *de novo* assembly of *S. homoeocarpa* and creeping bentgrass transcriptome libraries. SBS reads were mapped to the 454 assemblies to calculate transcript levels from *S. homoeocarpa* and creeping bentgrass during dollar spot disease development. The objective of this study was to identify transcripts that may be important for fungal virulence and creeping bentgrass defense. The results of the analysis will be used to form testable hypotheses for future studies on dollar spot etiology and turfgrass defense mechanisms.

## Results

### Transcriptomic Analysis

The *S. homoeocarpa* (SH) 454 sequencing data contained 600,760 reads total, and the *A. stolonifera* (AS) 454 sequence data contained 205,403 reads total (Table 1). The 454 read lengths ranged from 50 bp to >500 bp, with a majority of the reads between 400 and 500 bp in length. The transcriptome coverage for each of the 454 assemblies was 3.3× coverage for AS and 17.2× coverage for the SH assembly. These sequencing coverages were calculated by dividing the total number of sequence reads by the size of the respective, assembled transcriptome libraries.

**Table 1:** Characteristics of the 454 RNA-Seq data.

Transcript Assembly	Sequencing Libraries Included	Number of Reads	Number of Isotigs	Mean Isotig Length (bp)	Number of Singletons
**Total SH**	SH PDA 96h	600,760	10,101	1,172	51,502
	SH PDB 48h/96h/144h				
	Interaction 96h				
**Total AS**	AS 966h	205,403	5,017	898	58,446
	Interaction 96h				

The SBS data, which was used for calculating significant differences in transcript levels between libraries, resulted in 4.3–7.2 million reads (Table 2); however, these reads were only 16 bp long. This is due to the use of first generation Illumina sequencing protocols. The SBS reads provided ample coverage of the entire transcript assemblies: 14× coverage for SH and 12× for AS. The SBS library construction resulted in 9, 319 SH transcripts with lengths ranging from 400 bp to 3, 500 bp and a distribution peak at 500 bp. Construction of the SBS transcript library for AS resulted in 20, 293 transcript sequences with lengths ranging from 200 bp to 8,500 bp and distribution peak at 480 bp. The full length of transcripts was not estimated since very few annotated coding sequences from either SH or AS were available at Genbank. The mapping percentage of SBS transcripts was quite low. Only 67.4% of SH PDA reads, 63.8% of SH PDB reads, and 33.3% of Interaction reads were mapped to the SH transcripts assembly, respectively (Table 2). Only 16.8% of AS only reads, and 5.7% of Interaction reads mapped to the AS transcripts library (Table 2). This is likely due to the short length of the SBS reads and stringent mapping parameters used in this experiment, which eliminated reads mapping to more than one location and did not allow for any base mismatches.

**Table 2:** Mapping characteristics of the *Sclerotinia homoeocarpa* (SH) and *Agrostis stolonifera* (AS) SBS reads to the SH and AS transcript assemblies.

SBS Library	SH PDA	SH PDB	AS 96h	Interaction
Number of Reads	6,101,988	4,350,510	6,885,250	7,182,868
Mapping Percentage to SH Assembly	67.4%	63.8%	0.9%	33.3%
Mapping Percentage to AS Assembly	2.6%	2.4%	16.8%	5.7%

**Figure 1:**
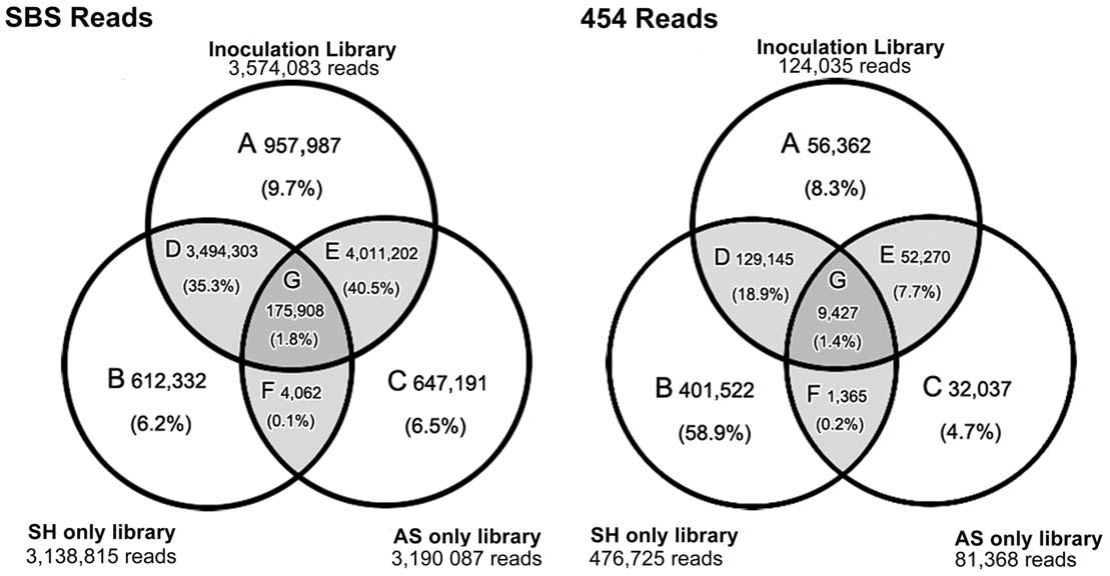
Venn diagram of RNA-Seq reads unique and common to the SH library, AS library, and Interaction library. A. Reads unique to the Inoculation library, B. Reads unique to the SH only library, C. Reads unique to the AS only library, D. Reads common to the Inoculation and SH library, E. Reads common to the Inoculation and AS library, F. Reads common to the SH and AS libraries, G. Reads common to the Inoculation, SH, and AS libraries.

A Venn diagram was constructed to identify the distribution of the unique and common reads in both SBS and 454 SH, AS, and interaction libraries (Figure 1). There are discrepancies in the read distribution that appear between the SBS and the 454 libraries. The most obvious example is the increase in the number of reads that are unique to the SH-only library from 6.2% in the SBS to 58.9% in the 454 data. This is because there were more total SH reads in the 454 dataset, where four of the six libraries (69.8% of the reads) were constructed using RNA from *S. homoeocarpa* grown in pure culture. In the SBS dataset, only two out of four (31.7% of the reads) of the libraries were constructed from *S. homoeocarpa* grown in pure culture. The number of reads common to the interaction and control libraries was consistently low in both SBS and 454 datasets (Figure 1). The common reads are likely a result of similar sequences in both organisms, including those of transposable elements found in both organisms.

### Comparative analysis

**Figure 2:**
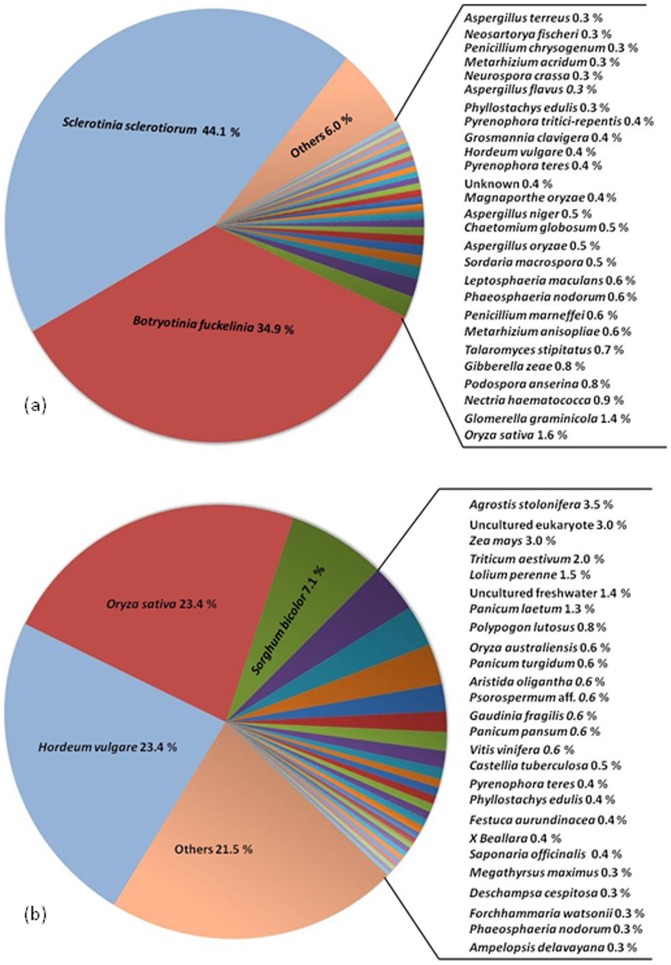
Top Species Blast hits: (a) SH transcript library (b) AS transcript library.

The transcripts were blasted against the NCBI protein database for functional annotation using Blast2GO [20] blastx function. Fifty seven percent (5,257) of the 9,319 SH transcripts returned at least one annotation hit. Likewise, 57% (11, 551) of the 20, 293 AS transcripts returned at least one hit. From the blastn taxonomy report, top hits of the *S. homoeocarpa* sequences were primarily from *Sclerotinia sclerotiorum* (Lib.) de Bary (44. 1%) and *Botryotinia fuckeliana* (de Bary) Whetzel (34.9%) (Figure 2 a). This is not surprising considering their taxonomic similarity to *S. homoeocarpa*, and the abundance of *S. sclerotiorum* and *B. fuckeliana* sequence data available on GenBank. The 26 fungal species represented in the taxonomy report belong to various classes of the subphylum Pezizomycotina, with seven of the 26 species being pathogens of monocot plants. There were a small number of sequences related to monocots including rice (*Oryza sativa* Linnaeus, 1.6%), bamboo (*Phyllostachys edulis* (Carr.) Houz., 0.3%), and barley (*Hordeum vulgare* Linnaeus, 0.4%). We attributed this either to contamination of the fungal sequences by *A. stolonifera* sequences in the interaction library, to the presence of very highly conserved sequences, or to the presence of sequences occurring in both organisms, such as those of transposable elements. The top blast hit species for the *A. stolonifera* sequences included predominantly monocot grass species (Figure 2 b). Fungal sequences that were found included those of *Pyrenophora teres* Smed.-Pet. and *Phaeosphaeria nodorum* (E. Müll) Hedjar, pathogens of barley and wheat, respectively.

### Comparison of RPKM ratios to Relative Transcription ratios

**Figure 3:**
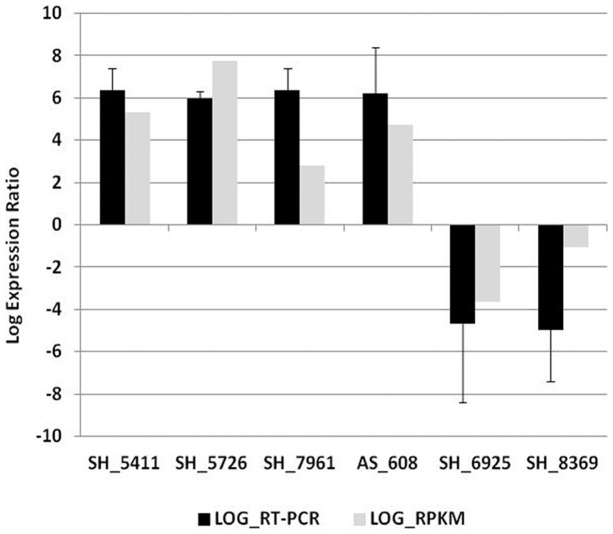
Validation of RPKM data for selected SH and AS transcripts using relative expression real time PCR.

Reads Per Kilobase of exon model per Million mapped reads (RPKM) value was calculated of all SH and AS transcripts in different libraries and the Log_2_ ratio was calculated to reflect the fold changes of interaction library compared to control libraries (SH PDA and AS only). Relative transcription data from real-time RT-PCR assays was obtained for selected transcripts with significantly different transcription levels during infection. The relative transcription data verified that the RPKM calculating and statistical analysis of transcriptome data was accurate (Figure 3). The real time RT-PCR data confirms the upregulation of two fungal xylanases (SH_5411 and SH_5726), a fungal laccase (SH_7961), and a creeping bentgrass germin protein (AS_608) (Figure 2). Downregulated transcripts SH_6925 (scyatalone dehydrogenase) and SH_8369 (β-mannosidase) were also verified (Figure 2).

### Transcripts Expression Analysis

To identify transcripts that were up-regulated in the interaction condition, transcripts with statistically significant changes of RPKM values during infection were examined to determine if there were patterns in the types of genes with increased or decreased transcription level. Table 3 summarizes some of the types of SH transcripts of interest during infection including a long list of glycosyl hydrolase transcripts and various proteases. Table 4 summarizes transcripts of interest in the AS library including a long list of retrotransposons, defense-related proteins including 13 different germin-like proteins, and various enzymes involved in plant hormone synthesis and regulation. Interestingly, all jasmonate-induced protein transcripts were significantly down regulated at 96 hpi. A full list of SH and AS transcripts that are significantly over or under expressed during infection are available as supplementary data (Tables S1 and S2, respectively).

**Table 3:** Summary of select *Sclerotinia homoeocarpa* transcript types that are significantly increased at 96 h post inoculation on creeping bentgrass.

Transcript Types	Description	Transcripts	Log fold change
**Glycosyl hydrolases**	ferruloyl esterase	2	4.8–11.5
	dihydroceramidase	1	3.1
	amylase	1	3.0
	arabinofuranosidase	7	2.4–9.9
	rhamnosidase	1	2.1
	endoglucanase	4	2.9–5.7
	glucosidase	4	3.1–4.9
	xylosidase	5	2.6–5.3
	cellobiohydrolase	4	4.3–6.9
	cellobiose dehydrogenase	1	4.1
	cellulase	1	3.6
	cutinase	1	3.0
	mannosidase	2	3.1–4.6
	xylanase	3	4.6–8.5
	glucanase	8	2.2–7.3
	polygalacturonase	3	3.0–8.1
	rhamnogalacturonase	2	2.9–8.1
	lysozyme	1	5.7
	pectinesterase	1	2.0
	**Total**	**52**	**2.0–11.5**
**Proteases**	serine protease	4	2.9–9.4
	aspergillopepsin-2	1	2.3
	peptidases	8	2.4–6.5
	neutral protease	1	5.8
	**Total**	**14**	**2.3–9.4**
**Transporters**	mdr-like abc transporter	7	4.1–6.6
	mfs multidrug	2	2.7–2.9
	**Total**	**9**	**2.7–6.6**

**Table 4:** Summary of select *Agrostis stolonifera* transcript types that are significantly increased at 96 h post inoculation with *Sclerotinia homoeocarpa.*

Transcript Types	Product	Transcripts	Log fold change
**Transposons**	athila retroelement	1	5.8
	copia-type retroelement	2	2.6–4.3
	gag-pol polyprotein	10	2.4–4.9
	mutator-like transposase	1	4.6
	retroelement pol poly	2	2.2–2.7
	retrotransposon line	1	3.0
	retrotransposon ty1-copia	6	3.4–5.8
	retrotransposon ty3-gypsy	13	2.5–8.6
	transposon en spm	15	2.0–5.2
	unidentified transposon	2	2.3–3.7
	retrotransposon unclassified	18	2.4–7.6
	**Total**	**72**	**2.0–8.6**
**Defense**	anthranilate synthase	1	4.5
	calcineurin	1	3.5
	cinnamoyl reductase	1	3.5
	cytochrome p450	5	2.0–5.5
	disease resistance nbs-lrr	3	2.3–4.0
	e3 ubiquitin-protein ligase	2	3.7–4.0
	fusarium resistance, i2c-5-like	1	2.9
	germin a	13	3.4–6.2
	mdr abc transporter	6	5.9–7.7
	pathogenesis associated pep2	1	9.1
	rust resistance kinase lr10	1	4.5
	terpene synthase	1	2.9
	ubiquitin	2	2.1–4.8
	ubiquitin-conjugating enzyme	1	2.1
	ubiquitin-specific protease	1	3.8
	zingiberene synthase	1	2.8
	**Total**	**47**	**2.0–9.1**

### Gene Ontology Analysis

**Figure 4:**
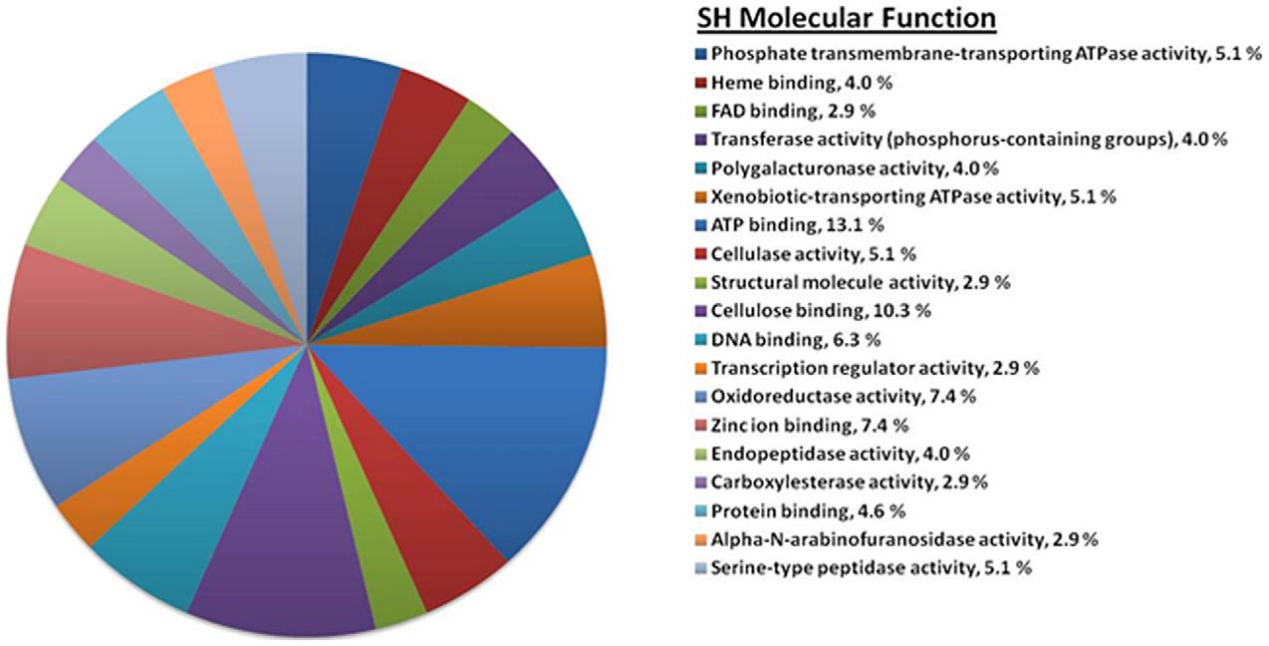
GO terms associated with upregulated SH transcripts. (a) Molecular Function, (b) Biological Process, (c) Cellular Component.

**Figure 5:**
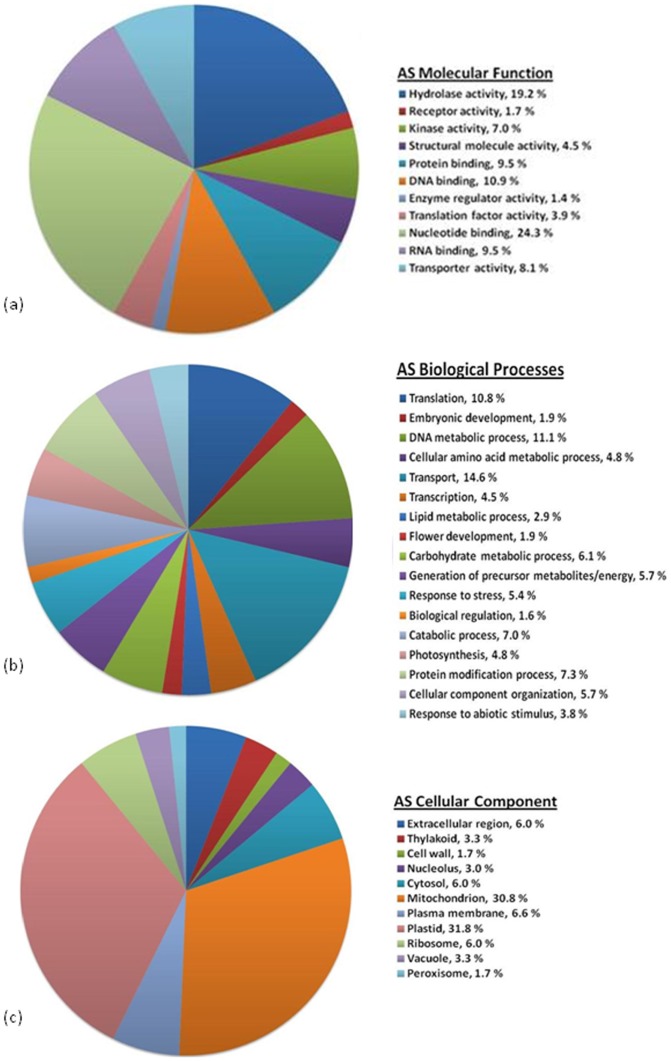
GO terms associated with upregulated AS transcripts. (a) Molecular Function, (b) Biological Process, (c) Cellular Component.

The list of significantly upregulated SH and AS transcripts were sorted into gene ontology (GO) term categories for molecular function, biological processes, and cellular component using the Blast2GO [20] program (Figures 4 and 5). The molecular function of GO terms associated with upregulated SH transcripts include a large percentage of plant cell-wall degrading enzymes categories (Figure 4 a) including polygalacturonase activity (4.0%), cellulase activity (5.1%), endopeptidase activity (4.0%), α-arabinofuranosidase activity (2.9%), and serine-type peptidase activity (5.1%). Other upregulated molecular function of GO terms included those for xenobiotic-transporting activity (5.1%) and oxidoreductase activity (7.4%). Biological process of upregulated SH transcript GO terms included auxin biosynthesis (11.4%), transmembrane transport (18.9%), and oxidation reduction (25.1%) (Figure 4 b). A majority of the upregulated SH cellular component GO terms were for those integral to the membrane (37.2%) and those for extracellular regions (34.0%) (Figure 4 c), perhaps reflecting the secretion of cell wall degrading enzymes and other secondary metabolites important for pathogenicity and virulence of *S. homoeocarpa*.

The molecular function of GO terms associated with the upregulated AS transcripts included those associated with hydrolase activity (19.2%), DNA binding activity (10.9%), and nucleotide binding (24.3%) (Figure 5 a). The biological process GO terms associated with the upregulated AS transcript library included those related to translation (10.8%), transport (14.6%), and DNA metabolic processes (11.1%) (Figure 5 b). Finally, an overwhelming number of cellular process GO terms from the AS upregulated library were associated with the mitochondrion (30.8%) and plastids (31.8%) (Figure 5 c).

### Conserved Domain Analysis

Protein domains were identified in the *S. homoeocarpa* and *A. stolonifera* transcripts libraries using HMMER software (v3.0) [21]. Also among SH transcripts, 1588 were predicted to contain signal peptide by signalP (v4.0) [22], and thus could be candidates for secreted proteins. Three proteases with secretion signals included two subtilisin/sedolisin proteases with log-fold increases of 6.4 and 6.2 as well as a cuticle degrading serine protease with a log-fold increase of 4.0. Conserved domains significantly enriched in the interaction library (*P*≤0.01) are listed in Table 5 (SH conserved domains) and Table 6 (AS conserved domains). Domains enriched in the SH interaction library include a variety of glycosyl hydrolases, proteases domains, and transporters (Table 5). Domains of interest in the enriched AS interaction library include cytochrome P450, various ABC transporters, and cupin domains, which include germin-class enzymes (Table 6). A complete list of SH and AS conserved domains can be found as supplementary data in tables S3 and S4, respectively.

**Table 5:** Top enriched *Sclerotinia homoeocarpa* domains at 96 h post inoculation on creeping bentgrass.

Domain name	Enrichment	P-value	Description
Glyco_hydro_45	17.0	0.0030	Endoglucanase
Glyco_hydro_62	17.0	0.0030	Alpha -L-arabinofuranosidase
Glyco_hydro_61	15.3	<0.0001	Endoglucanase
Peptidase_S28	13.6	0.0010	Serine carboxypeptidase S28
CBM_1	13.4	<0.0001	Fungal cellulose binding domain
E1_DerP2_DerF2	12.8	0.0003	Lipid recognition, recognition of pathogen products
Pectinesterase	12.8	0.0003	Pectinesterase
Eno-Rase_NADH_b	12.2	0.0003	NAD(P)H binding domain, trans-2-enoyl-CoA reductase
Glyco_hydro_43	11.4	<0.0001	Arabinanase
COX3	11.4	0.0016	Cytochrome c oxidase subunit III
Flavodoxin_2	11.4	0.0016	Flavodoxin-like fold
Glyco_hydro_11	11.4	0.0016	Xylanases
Glyco_hydro_12	11.4	0.0016	Endoglucanase and xyloglucan hydrolase
Nucleoplasmin	11.4	0.0016	Chromatin decondensation proteins
PLA2_B	11.4	0.0016	Lysophospholipase catalytic domain
Glyco_hydro_28	11.0	<0.0001	Polygalacturonase, Rhamnogalacturonase A
ABC_ATPase	10.8	<0.0001	Predicted ATPase of the ABC class
Syja_N	10.2	0.0006	SacI homology domain
Cellulase	9.7	0.0023	Cellulase
CFEM	9.7	0.0023	Cysteine rich, putative role in fungal pathogenesis
Flavodoxin_5	8.5	0.0030	Flavodoxin
FMN_red	8.5	0.0030	NADPH-dependent FMN reductase
Glyco_hydro_7	8.5	0.0030	Endoglucanase; cellobiohydrolase
Glyco_hydro_92	8.5	0.0030	Alpha-1,2-mannosidases
Mannosyl_trans3	8.5	0.0030	Mannosyltransferase putative
Yos1	8.5	0.0030	Transport between ER and the Golgi complex
7tm_1	8.5	0.0089	7 transmembrane receptor
Chorismate_synt	8.5	0.0089	Chorismate synthesis
COX6B	8.5	0.0089	Cytochrome oxidase c subunit VIb
Cu-oxidase_2	8.5	0.0089	Multicopper oxidase
Cupin_5	8.5	0.0089	Cupin superfamily
DHDPS	8.5	0.0089	Dihydrodipicolinate synthetase family
Fig1	8.5	0.0089	Ca2+ regulator and membrane fusion protein Fig1
Metallophos_2	8.5	0.0089	Calcineurin-like phosphoesterase
Pex2_Pex12	8.5	0.0089	N terminal of a number of known and predicted peroxins
Ribosomal_L35Ae	8.5	0.0089	Ribosomal protein L35Ae
SAPS	8.5	0.0089	SIT4 phosphatase-associated protein
UbiA	8.5	0.0089	UbiA prenyltransferase family
ABC_membrane	8.2	<0.0001	ABC transporter transmembrane region
Fungal_lectin	6.8	0.0061	Fungal fucose-specific lectin
OPT	6.2	0.0080	OPT oligopeptide transporter protein
Peptidase_S8	6.1	<0.0001	Subtilase family
ABC_tran	5.8	<0.0001	ABC transporter

**Table 6:** Top enriched *Agrostis stolonifera* domains at 96 h post inoculation with *Sclerotinia homoeocarpa.*

Domain name	Enrichment	P-value	Description
eIF-5_eIF-2B	20.2	<0.0001	Zinc binding C4 finger.
ATS3	20.2	0.002	Embryo-specific protein 3
BDS_I_II	20.2	0.002	Antihypertensive protein BDS-I/II
CSD	20.2	0.002	‘Cold-shock’ DNA-binding domain
Folate_rec	20.2	0.002	Folate receptor family
MaoC_dehydratas	20.2	0.002	Synthesis of monoamine oxidase
SH3_1	20.2	0.002	Signal transduction related to cytoskeletal organisation
TAFII28	20.2	0.002	Assembly of the transcription preinitiation complex.
ABC2_membrane_3	15.1	<0.0001	ABC-2 type transporter
Fer4	13.5	<0.0001	Iron sulfur binding domain
Fer4_2	13.5	<0.0001	Iron sulfur binding domain
Fer4_6	13.5	<0.0001	Iron sulfur binding domain
Ribosomal_S2	13.5	<0.0001	Ribosomal protein S2
CALCOCO1	13.5	0.0077	Calcium binding and coiled-coil domain (CALCOCO1)
iPGM_N	13.5	0.0077	independent phosphoglycerate mutase
tRNA-synt_1c	12.6	<0.0001	tRNA synthetases class I (E and Q), catalytic domain
TMF_DNA_bd	12.1	0.0004	TATA element modulatory factor 1 DNA binding
UDPGT	12.1	0.0004	UDP-glucoronosyl and UDP-glucosyl transferase
Cupin_1	11.9	<0.0001	Cupins, including germins
Cupin_2	11.1	<0.0001	Cupin domain
ABC2_membrane	10.9	<0.0001	ABC-2 type transporter
GrpE	10.1	0.0011	GrpE nucleotide exchange factor
EFG_C	9.0	0.0002	Elongation factor G C-terminus
GTP_EFTU_D3	8.8	<0.0001	Elongation factor Tu C-terminal domain
ABC_ATPase	8.7	<0.0001	Predicted ATPase of the ABC class
IF-2B	8.7	0.0025	Initiation factor 2 subunit family
RNA_pol_Rpb1_5	8.7	0.0025	RNA polymerase Rpb1, domain 5
Cytochrom_B_C	7.6	0.0049	Cytochrome b/b6, C-terminal
SMC_N	7.3	<0.0001	structural maintenance of chromosomes protein
ABC_membrane	6.7	<0.0001	ABC transporter transmembrane region
ATP-synt_C	6.7	0.0086	ATP synthase subunit C
ATP-synt_ab_C	6.7	0.0012	ATP synthase alpha/beta chain
DUF1602	6.1	<0.0001	Protein of unknown function
GTP_EFTU	5.8	<0.0001	Elongation factor Tu GTP binding domain
ABC_tran	5.5	<0.0001	ABC transporters
Terpene_synth_C	5.4	0.0049	Terpene synthase family, metal binding domain
p450	5.0	<0.0001	Oxidative degradation of compounds
GTP_EFTU_D2	5.0	0.0005	Elongation factor Tu domain 2

## Discussion

Previous analyses using RNA-Seq have evaluated the reproducibility and accuracy of SBS [18] and 454 data for RNA transcription studies [23]. Both technologies have demonstrated higher-quality results than microarray and EST data [18], [19]. However, SBS technologies were found to be superior due to the greater coverage and depth of sequences for a much lower cost. In this study, the mapping percentage of the SBS data to the *S. homoeocarpa* assembly ranged from 33.3–67.4% and *A. stolonifera* mapping percentages ranged from 5.7–16.8%. The mapping percentages were low due to the very stringent parameters that eliminated all reads with even a single mismatch and reads that mapped to more than a single location, as well as possible transcriptome misassembly. The use of these parameters and their resulting influence on the statistical analysis of the RPKM data was verified with real-time relative expression data. Mapping uncertainty in RNA-Seq analyses is one of the pitfalls of the SBS sequencing method. Short sequence lengths, as was the case in this study, make it difficult to account for paralogous genes, alternatively spliced isoforms, and low complexity sequences, all of which result in multireads that are eliminated in the mapping process [24]. Low mapping percentage may also be due to the lower coverage of 454 transcriptome assemblies compared to the high SBS coverage.

RNA-Seq analysis in this study has made it possible to identify potential pathogenicity factors transcribed by *S. homoeocarpa* during infection and colonization of *A. stolonifera*. The most striking discovery was the number of enriched *S. homoeocarpa* transcripts within the interaction library that encoded glycosyl hydrolase enzymes. In fact, 52 of the upregulated SH transcripts encoded glycosyl hydrolase genes and 22.3% of molecular function GO terms were associated with glycosyl hydrolase activity. Within the total SH transcript library, a keyword search using search terms such as glycosyl hydrolase, cellulase and pectinase revealed over 100 transcripts annotated as glycosyl hydrolases in the SH library, representing 85 different families of the total 125 glycosyl hydrolase families [25], [26]. The numerous transcripts representing such a varied group of glycosyl hydrolase families corroborate the ability of this fungus to infect and cause disease on a wide range of plant hosts. In this study, *S. homoeocarpa* glycosyl hydrolase genes showing upregulation were predominantly xylanases and arabinases. This is not surprising since grass cell walls consist of approximately 40% xylans as well as glucuronoarabinoxylans (GAX) that make up a majority of monocot hemicelluloses [27]. Enzymes such as pectinases, xylanases, and cellulases are often virulence and pathogenicity factors for plant pathogenic fungi and bacteria. For example, *Botrytis cinerea* Pers. and *Septoria nodorum* (Berk) Berk., demonstrate reduced virulence when a single gene encoding a cell wall degrading enzyme is knocked-out [28], [29], [30]. However, the large number of upregulated and functionally redundant cell wall degrading enzymes expressed by *S. homoeocarpa* interacting with creeping bentgrass will make verifying the role of any one enzyme extremely difficult.

Numerous proteinases, mostly serine proteases, were also enriched in the fungal interaction library. Secretion signals were detected on three of the proteases of interest, including two subtilisin/sedolisin proteases and one identified as a cuticle degrading serine protease. These three secreted proteases were upregulated in the interactoin library by 4.0–6.4 log-fold. Of the molecular function GO terms, 5.1% were associated specifically with serine-protease activity. Serine proteases are pathogenicity determinants for a variety of pathogenic fungi including nematode-parasitizing fungi, entomopathogens, and plant pathogenic fungi [31]. For example, protease deficient mutants of the hemibiotroph *Pyrenopeziza brassicae* B. Sutton & Rawl. were either avirulent or had dramatically reduced virulence [32]. Proteases in other plant pathogenic fungi have been characterized, but their role in pathogenicity has not necessarily been confirmed in each case [33], [34].

In general, the presence of a variety of hydrolytic enzymes including glycosyl hydrolases and proteinases supports the broad host range reported for *S. homoeocarpa* [1]. The diversity and number of enzymes also support saprophytic ability of *S. homoeocarpa* [35]. For example, fungi with superior saprotrophic abilities, such as *Aspergillus* spp. were found to produce a more diverse collection of hydrolytic enzymes under a variety of conditions, while phytopathogens with inferior saprophytic abilities such as *Verticillium albo-atrum* Reinke & Berthold and *V. lecanii* (Zimm.) Viégas, produced a more targeted array of enzymes suited to their particular host range [35]. The large number of hydrolytic enzymes expressed by *S. homoeocarpa* supports the hypothesis of superior saprophytic abilities of the dollar spot pathogen, which may play a role in the overwintering of the fungus within the thatch during the winter months.

Multi-drug resistance (MDR) ATP binding cassette (ABC) transporter proteins were also upregulated in the *S. homoeocarpa* interaction library, with seven of the MDR-ABC transporters being upregulated while only one MDR-ABC transporter transcript was downregulated. Members of the MDR-ABC transporter family have important roles in fungicide resistance, resistance to plant defense compounds and efflux of endogeneous toxins [36], [37]. In *B. cinerea*, both ABC and MFS transporters have been implicated in resistance of the pathogen to a variety of fungicides including phenylpyrroles, azoles, anilopyrimidines, dicarboximides, and strobilurins [38], [39], [40]. The ABC transporter BcatrB of *B. cinerea* is strongly induced in the presence of fludioxonil, cyprodinil, cyproconazole, tebuconazole, and trifloxystrobin suggesting a role for this single transporter in resistance to multiple fungicide types [41]. Enrichment of a variety of transport proteins with similar function in the *S. homoeocarpa* interaction library may provide an explanation for the development of isolates that are resistant to more than one fungicide class. For example, isolates are known that are resistant to demethylation inhibitors as well as benzimidazoles and dicarboximides [1], [42], [43]. Aside from fungicide resistance, ABC transporters are also implicated in resistance to plant defense compounds and secretion of fungal toxins. For example, BcatrB not only provides resistance to fungicides but also to the plant defense compounds resveratrol and camalexin [39], [40]. Characterization of these transporter proteins could lead to a better understanding of fungicide resistance mechanisms and *S. homoeocarpa-*creeping bentgrass interactions, resulting in more effective fungicide application programs and fungicide resistance management strategies.

Integrated fungicide resistance management recommendations often include using cultivars of grass that have increased pathogen resistance. Several cultivars of creeping bentgrass have decreased susceptibility to dollar spot disease [44], [45]; however, the molecular defense mechanisms responsible for the decrease in susceptibility are unknown. In this study, some of the most prominent, upregulated defense-related transcripts were the germin and germin-like proteins. Germins belong to the cupin superfamily and include the important defense enzyme, oxalate oxidase [46]. Germin-type oxalate oxidases are enzymes that are unique to Gramineae plants [46]. In creeping bentgrass cultivars, oxalate oxidase has been shown to be more active in the moderately resistant creeping bentgrass cultivar, L-93, compared to Crenshaw, a highly susceptible cultivar [47]. Oxalic acid is a pathogenicity factor for *Sclerotinia sclerotiorum* (Lib.) de Bary [48], and oxalic acid is produced in large quantities by *S. homoeocarpa* as well [49]. Cloning oxalate oxidase into dicot hosts such as sunflower, soybean, canola, and tomato renders them resistant to *S. sclerotiorum* [50], [51], [52]. It is possible that increased production of oxalate oxidases by some cultivars of creeping bentgrass is responsible for their decreased susceptibility to *S. homoeocarpa*. Furthermore, the product of oxalate oxidase activity is H_2_O_2_, a plant defense signal that is capable of passing through cell membranes, is directly toxic to invading microorganisms, cross-links glycoproteins and phenolic molecules to form defense structures, and can induce systemic acquired resistance [53], [54]. Clearly, more research on germin-like proteins and oxalate oxidase activity in creeping bentgrass is warranted.

In addition to the plant defense-related transcripts, other notable creeping bentgrass transcripts were identified as transposons and retrotransposons including pong, em, mutator, gypsy, and copia subclasses. This is not surprising since it has been documented that transposons make up approximately 80% of the DNA in cereal crops [55]. In a recent EST analysis of creeping bentgrass and colonial bentgrass, 1.4% and 0.18% of the total ESTs were retrotransposon related, and retrotransposon transcripts were eight times higher in creeping bentgrass under disease stress than in healthy colonial bentgrass plants that were not under disease stress [56]. In the RNA-Seq data analysed here, 878 of 20,493 (4%) of the total AS transcripts were identified as transposons and 731 (3.5%) were identified as retrotransposons. Seventy-two of the 1017 transcripts upregulated in the interaction library were identified as retrotransposons: that is 7% of all upregulated AS transcripts. This is in clear support of the previous study concluding that retrotransposons in creeping bentgrass are likely activated by disease stress [56]. In the shared library (reads present in both AS and SH), only 0.1% of the reads mapped to transcripts identified as transposons. This means that it is not likely that identical transposon sequences are shared between SH and AS. However, several of the classes of retrotransposons identified in the study, such as the *Ty* and *copia* classes, are known to exist in both fungi and plants [57], [58]. The upregulation of 72 retrotransposons in this study provides direct support for activation of transposons in creeping bentgrass in response to disease stress as demonstrated previously [56].

It has been reported that retrotransposons are transcriptionally activated in plants that are undergoing stress such as during protoplast formation, wounding, and in the presence of salicylic acid and jasmonate [59]. The influence of retrotransposons, if any, on plant defense pathways has not yet been determined; however, their increased transcription during infection should be investigated. For example, it would be interesting to determine if any of these retroelements are located within or near genes involved in plant defense responses, where they could affect the expression of these genes.

The RNA-Seq analysis presented in this study demonstrates the immense value of MPS technology for developing a comprehensive understanding of disease pathosystems. Advances in SBS technology since the start of this study would now provide adequate read length and depth for the *de novo* transcriptome assemblies, making it even more powerful and cost efficient. The resulting data analysis will be used to support future research efforts to characterize *S. homoeocarpa* virulence factors and creeping bentgrass defense-related transcripts.

## Materials and Methods

### Preparation of RNA for SBS Libraries

*Sclerotinia homoeocarpa* isolate MB01 was isolated from creeping bentgrass at The Ohio State University Turfgrass Research and Education Facility in Columbus, Ohio. The fungus was cultured on potato dextrose agar (PDA, Difco, Franklin Lakes, NJ) and 5 mm plugs of actively growing mycelium were transferred to potato dextrose broth (PDB, Difco) medium and incubated at 26°C with shaking at 160 rpm for 96 h. Cultures grown on PDA overlayed with cellophane membranes were also used for SBS library construction. At 96 h, mycelia were filtered from PDB or scraped from the cellophane membrane on PDA, and total RNA was extracted using TriReagent (Sigma, St. Louis, MO) according to manufacturer's instructions. For interaction and creeping bentgrass libraries, creeping bentgrass cv. Crenshaw was grown in a growth chamber at 26°C day and 22°C night temperatures with a 12 h photoperiod and at 70% relative humidity. Three- week old seedlings were challenged with millet seeds colonized by *S. homoeocarpa* MB01. These plants were incubated in clear bags to create humidity for 48 h after which the plants were uncovered during the day and bagged again for the night. After 96 h, leaves from inoculated and noninoculated plants were harvested, homogenized in liquid nitrogen, and RNA was extracted using TriReagent (Sigma) according to the manufacturer's instructions.

The SBS library templates were prepared using the Illumina Duplex-Specific thermostable nuclease (DSN) normalization kit and were analyzed using the Illumina Genome Analyzer I (GAI) at the Molecular and Cellular Imaging Center of The Ohio State University in Wooster, Ohio. The resulting libraries included a 96 h PDB culture control, a 96 h PDA culture control, a 96 h creeping bentgrass control, and a 96 h *S. homoeocarpa* – creeping bentgrass interaction library.

### Preparation of RNA for 454 Libraries

*S. homoeocarpa* isolate MB01 was cultured on PDA and 5 mm plugs of actively growing mycelium were transferred to PDB medium and incubated at 26°C with shaking at 160 rpm for 48, 96, or 144 h. At each time point, mycelia were filtered and total RNA was extracted using TriReagent (Sigma) according to manufacturer's instructions. For interaction and creeping bentgrass libraries, creeping bentgrass cv. Crenshaw was grown in a growth chamber as described previously. Three-week old seedlings were inoculated in one of two ways: with a 5 mm plug of actively growing mycelium from a PDA culture or misting of a mycelia homogenate, in water, from a 96 h PDB culture of *S. homoeocarpa* MB01. These plants were incubated as described for SBS library preparation. After 96 h, leaves from inoculated and noninoculated plants were harvested, homogenized in liquid nitrogen, and RNA was extracted using TriReagent according to the manufacturer's instructions. Total RNA was sent to the Core Genomics Facility at Purdue University, Indiana for preparation and sequencing using Roche GS-FLX (454) sequencer with Titanium chemistry. Seven RNA-Seq libraries were prepared that included *S. homoeocarpa* PDB cultures at 48, 96, and 144 h after inoculation, *S. homoeocarpa* grown on PDA at 96 h, creeping bentgrass noninoculated controls, and for creeping bentgrass incubated for 96 h with *S. homoeocarpa* applied as a PDA culture plug or a mycelia homogenate in water.

### Sequencing, Assembly, and Library Construction

The 454 raw sequence reads were assembled using GS Data Analysis Software (v2.5, Roche, Indianapolis, IN) after removal of adaptor sequences. *S. homoeocarpa* (SH) reads from the PDA, PDB, and 96 hpi Interaction libraries, and the *A. stolonifera* (AS) reads from the 96 h control and 96 h Interaction libraries were assembled using GS *de novo* assembler (v2.5, Roche) with parameter setting as 90% identity and a minimum 40 bp overlap. To eliminate SH reads in AS assembly library, reads from the *S. homoeocarpa* (SH) Interaction libraries that matched creeping bentgrass (*Agrostis stolonifera*) GenBank EST data were removed from the SH transcript assembly. Then reads from the *A. stolonifera* (AS) Interaction library matching SH assembly were also removed. Both *S. homoeocarpa* and *A. stolonifera* transcriptome assembly as used in this study was submitted to GenBank TSA database (Accession: PRJNA84359).

Full length SBS reads were directly mapped to the SH and AS transcriptome libraries using the Maq (v 0.7) [60] alignment. Mapping parameters allowed for only unique mapped reads with no mismatches. The number of mapped SBS reads for each transcript was counted by “soap.coverage” in SOAPAligner package.

### Transcript Expression Analysis

RPKM values were calculated for each transcript by dividing number of mapped reads by length of the transcripts and number of total sequenced reads in this library. Fisher's Exact Test, a test used for analyzing unreplicated transcript data [61] was used to determine statistical significance of transcript RPKM value change. The test was done using a pairwise comparison of Interaction RPKM values versus RPKM from fungus grown on PDA, or for Interaction RPKM values versus uninoculated control grass RPKM values. The fungal data contained 9,319 unique annotated transcripts and the grass data contained 20,293 unique annotated transcripts. Therefore, the statistical significance of the Fisher's Test was evaluated against a Bonferroni corrected *P*-value of 1.86×10^−5^ and 2.43×10^−6^ for fungal and grass transcripts, respectively. Furthermore, only transcripts with at least 2-log fold change in transcript abundance were selected as upregulated. These conservative criteria were applied to avoid false positives.

The log-fold RPKM values of selected transcripts were validated using real time reverse transcription polymerase chain (RT-PCR) reaction on an iQ5 real time thermocycler (Biorad, Hercules, CA). RNA was extracted from fungus grown on PDA, noninoculated grass, and infected grass according to the previously described methods. RNA was extracted using Trizol (Invitrogen, Carlsbad, CA) and RNA extracts were treated with RQ1 DNAse (Promega, St. Louis Obispo, CA) according to the manufacturer's directions, except that RNase Inhibitor (Promega) was added to each 100 µl of RNA extract. Complementary DNA was created from 1 µg of total RNA using iScript (BioRad, Hercules, CA). PCR was conducted using the SYBR supermix (Biorad) as per manufacturer's directions. The cycle conditions were 95°C for 3 minutes followed by 40 cycles of 95°C for 30 s, 58.5°C for 30 s, and 72°C for 30 s. The melting temperature profiles and gel electrophoresis was used to evaluate the specificity of the reactions and the absence of primer dimers. Real time RT-PCR was conducted with three biological replicates for each of grass, interaction, and fungal cDNA samples. Three technical replicates of each cDNA sample were used in each experiment.

Reaction efficiency and relative expression data were analyzed using the relative expression software tool (REST) program (Qiagen; [62]). Log base two values of relative expression ratios calculated in the REST program and the corresponding log base two RPKM expression ratios were compared graphically.

### Library annotation

Taxonomic and functional annotation of the SH and AS transcripts were conducted by using Blast2GO [20] software to run blastx and blastn algorithms against non-redundant nucleotide/protein database from the National Center for Biotechnology Information (National Institutes of Health). Within the SH library, any transcripts that resulted in top blastn hit species of *Hordeum*, *Oryza*, *Sorghum*, *Agrostis*, or *Vitis* were removed. Similarly, any transcripts in the AS library that had top blastn hit species of *Sclerotinia*, *Botryotinia*, *Glomerella*, *Ajellomyces*, or *Nectria* were removed. Combined graphs for GO terms associated with statistically upregulated transcripts are presented in the results section.

### Conserved Domain Analysis

Transcript sequences were translated to proteins in all 6 possible frames, and conserved protein domains were identified using HMMER (v3.0) [21] to screen the Pfam-A database. All the predicted domains with E-value <0.01 were reported. There were a total of 14,161 domains identified in the SH transcripts assembly and 18,591 domains identified in the AS transcripts assembly. The enrichment ratio and significant value was calculated based on a Fisher's Exact Test. Descriptions for enriched domains were found on the Pfam website.

## Supporting Information

Table S1 **A complete list of** ***Sclerotinia homoeocarpa*** **transcripts that are over or under represented at 96 h post inoculation on creeping bentgrass.**
(XLSX)

Table S2 **A complete list of** ***Agrostis stolonifera*** **transcripts that are over or under represented at 96 h post inoculation with** ***Sclerotinia homoeocapra.***
(XLSX)

Table S3 **A complete list of** ***Sclerotinia homoeocarpa*** **predicted protein conserved domains.**
(XLSX)

Table S4 **A complete list of** ***Agrostis stolonifera*** **predicted protein conserved domains.**
(XLSX)
