# Correction: RNA-Seq Analysis of the *Sclerotinia homoeocarpa* – Creeping Bentgrass Pathosystem

**DOI:** 10.1371/annotation/36af97b5-137b-4629-946b-748b63438b03

**Published:** 2012-10-03

**Authors:** Angela M. Orshinsky, Jinnan Hu, Stephen O. Opiyo, Venu Reddyvari-Channarayappa, Thomas K. Mitchell, Michael J. Boehm

A portion of the image Figure 4 is missing. A complete, correct image of Figure 4 can be seen here: 

**Figure:**
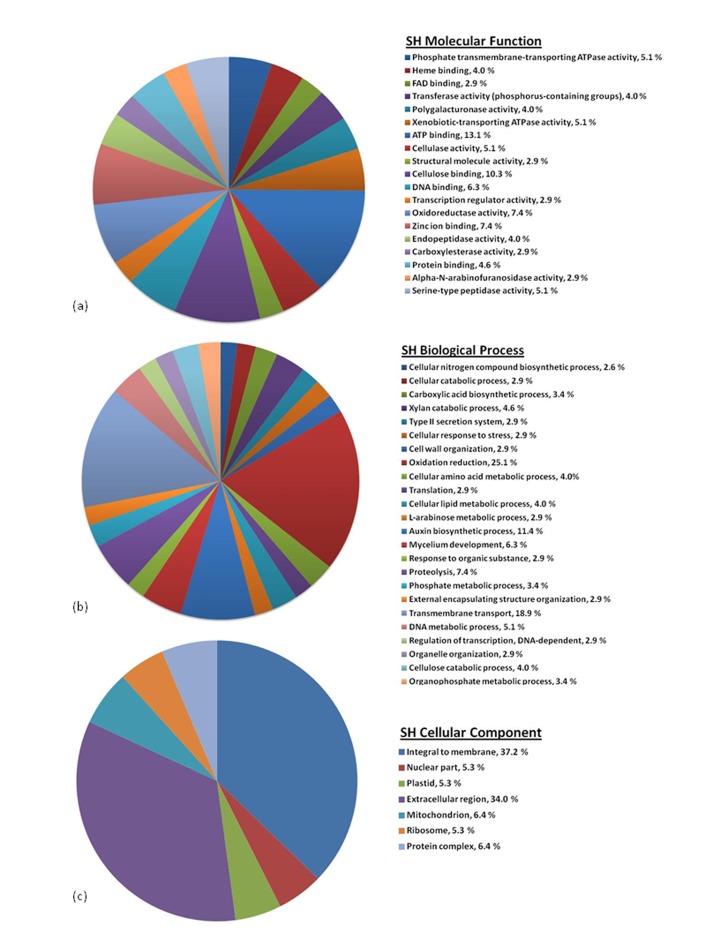


[^] 

